# Christmas and New Year “Dietary Titbits” and Perspectives from Chronobiology

**DOI:** 10.3390/nu14153177

**Published:** 2022-08-02

**Authors:** Thomas C. Erren, Ursula Wild, Philip Lewis

**Affiliations:** Institute and Policlinic for Occupational Medicine, Environmental Medicine and Prevention Research, Faculty of Medicine and University Hospital of Cologne, University of Cologne, Kerpener Straße 62, 50937 Cologne, Germany; tim.erren@uni-koeln.de (T.C.E.); ursula.wild@uk-koeln.de (U.W.)

**Keywords:** circadian, chronobiology, meal-timing, time-restricted feeding, intermittent fasting

## Abstract

A historical Christmas card connecting two pioneers of modern chronobiology (Colin Pittendrigh and Jürgen Aschoff) brings together key evolutionary facets of the field at Christmas time. The importance of the field to physiology and medicine is conveyed by the Nobel Prize award in 2017 for discoveries of how body clocks facilitate the temporal organization of physiology across days and nights. Temporal organization can have relevance for dietary Christmas excesses and dietary New Year resolutions. Herein, we examine how diet around Christmas and New Year has been targeted in human health research and we examine published opinion on dietary practice concerning Christmas and New Year using a systematized literature review approach. Thereafter, via a selective literature synthesis regarding time-restricted eating, we explore the chronobiological notion that “when” we eat and drink may make differences in terms of whether we experience weight gain and adverse health effects during and after the festive days. Overall, current Christmas eating is typically detrimental to health in terms of “how much” we consume of “what”. Regarding New Year’s goal-setting, chronobiology-based advice could be considered insofar as “when” we eat may be a healthier and more sustainable nutritional habit alternative. While we need further studies in humans, individual and public health may benefit during and after Christmas by adhering to plausible principles of chrononutrition. That detrimental nutritional excesses over Christmas may encourage individuals to tackle their eating habits should not be left untapped.

## 1. Introduction

**The timing makes the poison ....[1,2]**

-Variation of Paracelsus’ dictum:

*“What is there that is not poison?*

*All things are poison and nothing is without poison. Solely the dose determines that a thing is not a poison.”*

**Figure 1:**
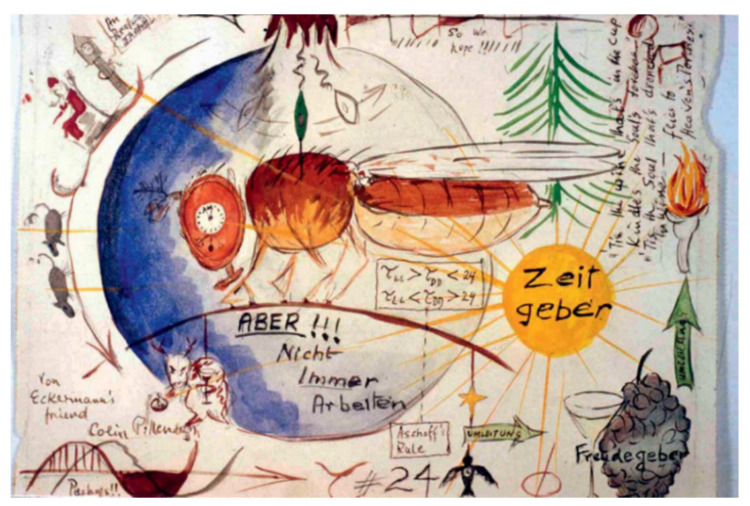
Christmas Card from Pittendrigh to Aschoff. Our thanks go to the late Gerta and Günther Fleissner for providing the photo.

A remarkable Christmas card was painted by Colin Pittendrigh for his long-term friend Jürgen Aschoff (Figure 1 [3])—both scientists were pioneers of modern chronobiology [4,5]. The card combines key facets of the field including our planet’s associated light-dark cycles as key Zeitgebers [6,7] or environmental time cues for circadian rhythms and endogenous clocks in fruit flies and birds—with Santa riding on his sled pulled by further study animals (rodents)—arranged around a backwards-ticking clock. The card’s centre shows a fruit fly with the brain’s central pacemaker, Aschoff’s far-reaching Zeitgeber concept, and a call for work–life balance: “Nicht Immer Arbeiten!” or “Don’t Always Work!”, which implies “Everything Needs Its Time”. The relevance of chronobiology may also be evinced by the 2017 Nobel Prize for discoveries how internal clocks temporally organise physiology and allow us to live in line with external rhythms of day and night [8]. Importantly, Pittendrigh exploring inner workings of body clocks in fruit flies [9,10,11] and Aschoff investigating links between Zeitgebers and circadian systems in rodents and birds [6,7] (all on Figure 1) contributed to paving the way for this Nobel Work [12].

Recently, nutrition has been placed under a microscope as a potential Zeitgeber candidate [13] and as an exposure to which the physiological response can depend on the phase of circadian clocks [14,15]. Indeed, beyond “how much” and “what” we eat, the “when” may significantly impact our health and disease risk via circadian systems [14,16]. This has been authoritatively conceptualized by Asher and the late Sassone-Corsi [17] as “chrononutrition”: “Chrononutrition refers to food administration in coordination with the body’s daily rhythms. This concept reflects the basic idea that, in addition to the amount and content of food, the time of ingestion is also critical for the well-being of an organism” [14].

Since the festive days over Christmas and New Year are times when people celebrate with food and drink and develop good intentions for the following year as a “fresh and healthy start”, we posed two questions: (i) What do scientific studies into Christmas and New Year, nutrition, health and disease tell us? (ii) What could be beneficial effects of considering chrononutrition over, and after, Christmas?

## 2. Objectives

Starting from Pittendrigh’s Christmas card (Figure 1 [3]) and with the festive days lying ahead, Part (i) examines how diet around Christmas and New Year has been targeted in human health research and we examine published opinion on dietary practice concerning Christmas and New Year using a systematized literature review approach. Moreover, Part (ii) is a selective literature synthesis, separate from the systematized approach of Part (i), that synthesizes how health benefits at and after Christmas might be achieved by considering research regarding chrononutrition (i.e., “when” we eat) via a selective literature synthesis. A concise discussion of Parts (i) and (ii) closes this paper.

## 3. Materials and Methods

For Part (i), the first step of zoning in on the relevant literature pool involved input of search terms pertaining to Christmas and nutrition combined with Boolean operators to the PubMed and Web of Science Core Collection literature search engines on 21 March 2022. The search string is presented in Table 1. The second step included title and abstract screening to rule out irrelevant literature based on whether articles fit our inclusion/exclusion criteria (see Table 1). The third step involved screening full texts to rule out irrelevant literature based on whether articles fit our inclusion/exclusion criteria. Inclusion/exclusion criteria are presented in Table 1. We exclude descriptive studies of alcohol consumption associated with traffic accidents, and hospital admissions as these are acknowledged occurrences which can be attributed to alcohol without a specific relation to Christmas. We also exclude primary research where health changes are described but dietary practice is only speculated about. Anecdotal evidence and opinion about dietary practice in non-primary research are included. Christmas during polar expeditions from 100 years ago was explicitly added as an exclusion criterion because an initial check yielded such studies but they lacked relevance to modern Christmas dietary practices en masse [18]. Data relevant to our aim were extracted and presented together in a cohesive synthesis.

For Part (ii), separate from the systematized approach of Part (i), we synthesized selected publications into links between calorie restriction, nutritional content, and the timing of food, on the one hand, and health and disease effects, on the other.

## 4. Results

### 4.1. Part (i) Systematized Literature Review Synthesis

**Figure 2:**
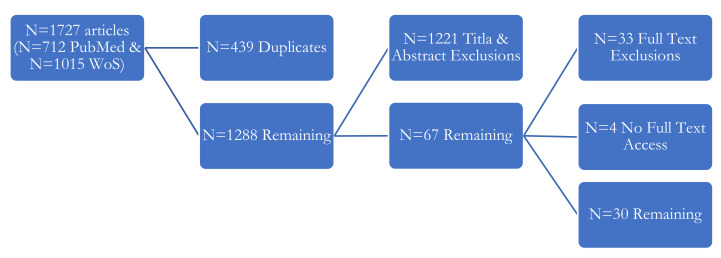
Modified PRISMA flow diagram [35].

The flow of articles through the various screening steps is illustrated in Figure 2. Briefly, our search string returned *n* = 1727 results. Following screening, *n* = 30 articles were determined as relevant (*n* = 17 primary source articles; *n* = 13 review/opinion style articles). Information from these articles pertaining to our aim is tabulated alongside general description of said articles in Table 2 (primary source articles) and Table 3 (review/opinion style articles).

Within the primary research, assessment of dietary consumption or changes thereof (or proxies such as purchases [19,20], visiting restaurants [21], or attending other social events [22]) were considered for descriptive assessment and as explanatory factors or endpoints in observational studies [20,21,22,23,24,25,26,27,28,29,30] or as an intervention in trials [31,32,33,34]. Wherein dietary practice was not the endpoint, various cardiometabolic parameters [21,22,27,28,29,30,31,32,34], acute symptoms [33], myocardial infarction [23], and clinical complexity of hospitalised patients [24] were investigated. Alcohol consumption per se was investigated in one study of college students and compared to other times of year [26]. Fasting was assessed in four articles that include a Greek Orthodox population from Crete for whom such fasting is a way of life and cardiometabolic parameters were compared to other times of year and to matched, non-fasting controls [27,28,29,30].

The outcomes were mostly as might be expected. More frequent diets and dietary behaviours that involve excess or food of lower nutritional value are reported around Christmas and New Year and these are associated with less healthy endpoint values or no change. One exception is a case–control study of myocardial infarction (cases = admitted to hospital, controls = chronic coronary syndrome but not admitted to hospital) wherein the controls had a higher increase in sweets consumption. This might seem counter-intuitive but could be due to reverse causation (i.e., those hospitalised consume less sweets during their stay) [23].

In terms of contributors to dietary practice around Christmas and New Year, social norms and subjective well-being appear to play a role with social norms changing between pre-Christmas, during Christmas, and post-Christmas [25].

Two trials targeted a prevention in weight gain [31,32]. One dietary supplement-supported intermittent fasting program (fasting 2 days per week for 6 weeks) did not cause a change in weight that was different from controls although a decrease from pre-Christmas to post-Christmas was observed [31]. One team-based weight-gain prevention program for 8 weeks found a decrease in weight from pre-Christmas to post-Christmas; however, weight increased above baseline by pre-Christmas in the following year [32].

Dietary practice at Christmas and New Year is, of course, different in different cultures [22,27,28,29,30,33]. Culture specific practices over the entire holiday period may differentially affect cardiometabolic parameters [22,27,28,29,30]. Acute cultural differences in consumption, such as concerning the Christmas day meal calorie and fat content, can also affect risk of reflux and dyspepsia [33].

Opinion articles and reviews concerning dietary practice over Christmas and New Year or that were stimulated by the time of year include variable content. For instance, they regarded interventions [36,37], trendy diets and warnings [38,39], New Year resolution practice [40], anecdotal evidence of specific dietary benefits [41], hypotheses regarding holiday-based weight gain and disease [42,43,44], points about health benefits of acute high calorie and moderate alcoholic celebration [45,46], and an ‘A-to-Z’ of dietary tips [47].

The opinion about interventions is more so that they are needed rather than providing specific information on how to achieve goals [36,37]. Some opinion regarding specific dietary practice and alcohol consumption should be carefully interpreted as it may now be out-dated (publications range from 1973 to present) [38,45,46]. Opinion that was perhaps more stimulated by the time of year rather than pertaining to the time of year per se appears in two essays by Cannon entitled ‘Out of the Christmas Box’. The author advocates researching the dietary practices of different cultures that have stood the test of time for potential value, such as fasting, and links with cultural contributions to Christmas, nutrition, and health in observational studies [22,27,28,29,30,33].

**Table 2:** Primary Research Targeting Diet at Christmas and New Year.

First Author (Year)	Study Design	Recruitment & Participants	How Was Diet Targeted?	Outcome
Kadhim (2021) [25]	Prospective longitudinal study	• *n* = 497 adult English native-speakers living in the U.S. participated in first wave of study. *n* = 318 participants completed the study. • Recruitment: crowdsourcing website.	• Questionnaire included asking about eating habits (unhealthy eating = rich in sugar, fats, or salt, and alcohol). • Questionnaire included asking about behavior, influence of social norms, and subjective well-being.	Over Christmas: • More unhealthy food consumption, • This behaviour was more normalised by peer groups, • More vitality, • No change in health perception. • Post-Christmas: • Perception of health and unhealthy eating was worse.
Olsson (2021) [23]	Case–control study	• *n* = 189 survivors of type I myocardial infarction (MI) at the Christmas days in Sweden. • *n* = 157 matched controls, diagnosed with stable angina and treated with percutaneous coronary intervention within 5-years prior to inclusion and who did not seek medical attention. • Recruitment: Database	• Questionnaire included asking about food and sweets consumption at Christmas.	• Food and sweets consumption was increased in both groups but more so in the control group.
Bhutani (2020) [21]	Prospective longitudinal study	• *n* = 23 independently living obese adults in the USA. • Recruitment: flyers and newspaper advertisements.	• Questionnaire about behavioural factors included dietary practice • Body weight change and endocrine parameters over the Christmas holiday period were also assessed.	• Body weight increased but there was no change in total energy expenditure. • More eating in restaurants was reported but no changes in other consumption habits (fast food, take-out, frozen meals). • No change in depression questionnaire or cortisol scores. • Meal satisfaction was lower after holiday period. • No change in appetite regulatory hormones was measured.
Cherchye (2020) [19]	Observational study	• *n* = 3645 British adults who live on their own. • Recruitment: data collected in the Kantar Wordpanel, which contains information on a representative sample of over 25,000 households.	• Purchases, food types, and calories were assessed. Grocery purchases defined as “healthy” or “unhealthy” (according to a nutrient profiling score) were tracked.	• Share of calories from healthy foods is highest in January and declines steadily over the year, reaching a low in December. • There is substantial heterogeneity in purchases across people, both in their average purchasing patterns and the extent to which their food choices vary over time.
Lenti (2020) [24]	Prospective, longitudinal study	• *n* = 106 cases were defined as patients hospitalised during mid Dec-Jan. • *n* = 121 controls were defined as patients hospitalised during mid Jan-Feb. • Recruitment: From an internal medicine unit in a hospital in Northern Italy.	• “Inappropriate diet”, was determined by trained data gatherers.	• Cases were older and had a higher clinical complexity (CC) score. • Inappropriate diet, inter alia, was associated with cases.
Hirsh (2019) [31]	Pilot randomized study	• *n* = 22 overweight healthy adults in the USA. • Recruitment: posters, flyers, social media, email blasts.	• Intervention was a dietary supplement-supported intermittent fasting programme (2 days per weeks) between Thanksgiving and New Year.	• No weight or weight loss differences between groups. • Differences in insulin, total:HDL cholesterol, alanine aminotransferase, and HOMA-2 were observed. • Pre vs. post intervention differences were observed for weight (↓), alanine aminotransferase (↑), HDL (↑), triglycerides (↓). • Some control group differences were also observed.
Wilson (2019) [32]	Pre/post study	• *n* = 97 state employees from one department (containing ~1000 people) in the USA were recruited.	• Specific team-based, weight-gain prevention program from Halloween until New Year. • Survey on eating behaviors was utilized.	• Mean weight decreased and healthy eating increased (increased fruit and vegetable consumption, decreased fast food and sugar-sweetened drink consumption). • However, in repeat participants, the baseline and end of program weights were higher for year 2 than for year 1.
Parker (2017) [33]	Cohort study	• *n* = 84 of all adult faculty and delegates attending a gastroenterology conference in Switzerland. • Recruitment: invitation to participate.	• All conference participants got the same menu on 4 consecutive days composed of a typical meal eaten during the festive season in different countries (with different calorie and fat content). • Reflux/dyspepsia were assessed by self-report.	• Odds of reflux/dyspepsia symptoms increased with higher calorie Christmas dinners.
Pope (2014) [20]	Randomized controlled trial	• *n* = 207 households who part of a larger seven-month study. • Recruitment: from three stores in West New York, through face-to-face public intercept, emails, word of mouth, and flyers.	• Food shopping transactions were recorded for 7 months and holiday season was compared to non-holiday season.	• Household food expenditure increased 15% during the holiday season with 75% of increased expenditure on less-healthy items. • Healthy food sales increased in the new year post-holiday season but unhealthy food sales remained high. • Estimated calories per serving was also higher in these periods.
Wagner (2012) [22]	Cohort study	• *n* = 34 non-student adults aged 23–61 years. • Recruitment: Word-of-mouth and from advertisements placed at businesses in this community of the USA.	• Dietary habits were assessed by questionnaire. • Weight and body composition were examined pre- and post-6 weeks that included Christmas and New Year.	• Number of days reported as over-eating was correlated with weight and BMI change. • Vegetable intake decreased while regular soda intake, “splurging” and social events increased. • There was no statistically significant change in weight.
Neighbors (2011) [26]	Survey	• *n* = 1124 undergraduate college students recently turned 21 years in the USA. • Recruitment: mail/e-mail.	• The study consisted of an online survey about alcohol consumption over previous 90 days. Consumption on each day was compared to 21st birthday, typical weekday, and typical weekend.	• Estimated mean blood alcohol levels given reported alcohol consumption were higher on New Year’s eve and day compared to non-holiday days. • Other days within this Winter holiday period were higher compared to non-holiday weekdays but lower than non-holiday weekends.
Sarri (2009) [27]	Case–control study	• *n* = 38 fasters during 3 holiday periods over 1 year (including Christmas). • *n* = 29 matched non-fasters. • Recruitment: from an adult population of Greek Orthodox Christians on Crete.	• Fasters during Christmas holiday period were compared to matched non-fasters.	• Fasters had reduced retinol and α-tocopherol and non-fasters had increased parameters but all were above safety limits.
Sarri (2007) [28]	Case–control study	Same as study above.	Same as study above.	• Fasters had generally higher blood pressure. • Fasting periods *per se* did not alter blood pressure. • The prevalence of high blood pressure decreased in the non-fasting group over Christmas. • Sodium and Calcium decreased in fasters but increased in non-fasters.
Sarri (2004) [29]	Case–control study	• *n* = 60 fasters during 3 holiday periods over 1 year (including Christmas). • *n* = 60 non-fasters. • Recruitment: selected from an adult population of Greek Orthodox Christians on Crete.	Same as study above.	• Pre vs. post-Christmas intake in energy, calcium, cholesterol decreased in fasters compared to non-fasters in addition to % energy from fat, saturated fatty acid, and monounsaturated fatty acid. • The % energy intake from carbohydrates increased. • There were no differences in fibre, protein, or polyunsaturated fatty acid (% energy). • Fasting generally was associated with differences in nutrient intake.
Sarri (2003) [30]	Case–control study	Same as study above.	Same as study above.	• Fasting was associated lower cardiometabolic parameters compared to non-fasters and pre-fasting. • Parameters increased during non-fasting periods as much as decreased during fasting periods (LDL, glucose, triglycerides, BMI, BP). • Macronutrient intake (fat, carbohydrate, protein, fibre) had also differentially changed between groups.
Cowley (1986) [34]	Clinical trial	• *n* = 6 healthy patients who were undergoing routine coronary arteriography for chest pain.	• Volunteers were assessed after a typical Christmas lunch (poultry, mince pies, and a glass of wine summing to 1400 kcal) in the UK. • The primary aim of the paper was to test feasibility of the technique.	• Cardiac output was measured by a CO_2_ rebreathing technique. “In all the subjects cardiac output was higher 15, 30, and 45 mins after the meal than before it, and the mean maximum increase was 30%. In one subject cardiac output had returned to the resting value 60 min after the meal, in the others it remained higher.” The volunteers considered the meal to modest in comparison what might be expected for Christmas.

The row colours add clarity by clearly separating different aspects or studies within the tables.

**Table 3:** Published Opinion Concerning Diet at Christmas and New Year.

First Author (Year) Article Type	Opinion (Direct Quote or Paraphrased)
Garrow (2000) [38] Comment	Dietary advice from many bestselling books at New Year—such as, e.g., that protein and carbohydrate should not be eaten together, the timing of meals should be altered, or unlimited quantities of particular foods to boost metabolism—have no scientific basis. Yet, their practice will be associated with some weight loss because dietary instruction generally causes a temporary decrease in total energy intake and there is a tendency to lose weight after Christmas anyway.
Zorbas (2020) [36] Review	This article is a scoping review on how festive feasting periods and celebrations contribute to population weight gain. The authors’ conclude that: “Interventions targeting festive periods could have a significant impact on population weight gain. The scalability and sustainability of such interventions require further investigation, as do the broader socioecological factors driving unhealthy eating during festive periods.”
Yeomans (2019) [40] Conference Paper	Dry January (temporary alcohol abstinence initiative) organised by Alcohol Concern in the UK attempts to regulate consumption by positive means (encouraging messages, reassurance against doubt, substitutes, promoting non-alcohol centred social integration). This is in comparison to doing something for others such as raising money for charity or by stigmatising alcohol consumers. Social media posts from the majority of study participants were positive and included mention of psychological, social, economic, and physical benefits.
Brendieck-Worm (2017) [39] Essay	Positive effects on blood glucose, triglycerides, total and LDL-cholesterol by cinnamon are put forward. On the other hand, that high cumarin levels in cinnamon biscuits may lead to consumption of higher than the recommended upper limit, especially for children, is indicated.
Bates (2016) [41] Anecdote	The author (a nurse) consumed turmeric, black pepper, ginger, cinnamon (presumably with honey in warm milk, presumably every day) and no longer feels the need to take anti-inflammatories. The spices were at the back of the authors cupboard and only taken out at Christmas time.
Eagle (2012) [42] Review	Increased catecholamine levels by, e.g., stress with diet-based inhibition of the SULT1A enzymes, possibly aided by genetic predisposition to SULT1A inhibition, may partly explain increased sudden cardiac death (SCD) around holidays (which still lacks a satisfactory explanation). This does not mean that the food supply is unsafe or that overindulgence in plant based foods and alcohols will lead to SCD. Yet, some people may need to moderate consumption of some foods and alcohols if combined with situations of stress or excitement.
Cannon (2006) [48] Essay	The essays by Cannon are published at Christmas time and they include the author’s reflections on dietary practice. In 2006, the author states: “The people of Zhejiang remain famous for their good health and long lives, and their diets now, which are still mostly traditional, meet the current WHO recommendations for dietary constituents and for vegetables and fruits”.
Cannon (2004) [49] Essay	The essays by Cannon are published at Christmas time and they include the author’s reflections on dietary practice. In 2004, the author states: “The value of fasting must be researched, as it is a relatively common practice that has persisted across centuries.”
Kloner (2004) [43] Editorial	The author considers that diet, among other factors, may be related to an increase in cardiac events at Christmas and New Year in the USA.
Harris (2003) [37] Editorial	Increasing fruit and vegetable intake should be a New Year resolution priority in Australia as a preventable contributor to disease: “Inadequate fruit and vegetable intake is responsible for approximately 3% of the disease burden.”
Clark (1998) [47] Poster	The author provides 26 snippets (from A to Z) of healthy eating tips for “A Nutritious New Year”. The 26 are too many to list here so we recommend viewing the article.
Griffith (1995) [45] Viewpoint	“We have a duty to tell our patients and the wider public what lifestyle changes may be beneficial to them. The benefits of a change to a regular moderate intake of alcohol are equivalent to giving up smoking and are far greater than regular exercise or diet. The collected evidence (more than five million subject-years follow up) shows that moderate drinking is of more benefit than perhaps any other intervention in cardiology. Our advice should be “consume one or two drinks a day, preferably with meals and perhaps red wine”. Patients already drinking at this level should be encouraged to continue, and lifetime teetotallers should be informed of the hazards of their continued abstinence. The hazards of heavy drinking should be highlighted and if necessary patients should be encouraged to cut their consumption.”
Hendry (1987) [44] Letter to Editor	“The report by Dr Cowley and colleagues (20/27 December, p 1422), in which they found an increased cardiac output after Christmas lunch, supports an observation which I have made at many necropsies. I have noted full stomachs in several elderly people with severe coronary artery disease who died of acute cardiac arrhythmia with no fresh occlusion. Such patients are at definite risk when a large meal is eaten, which substantially increases the work of the heart and tips a precarious cardiovascular balance. This danger is compounded when alcohol with the meal further increases the raised cardiac output.”—this is the letter in full.
Anonymous (1973) [46] Editorial	Referring to calorie loaded Christmas Pudding which is often eaten in the UK at Christmas: “It’s sustenance is of the spirit, not of the body. The mind is enriched and tranquil after such a meal, free to dream peace. Fully of those sunlit lands far over the blue, untaxable sea…. From hurrying to gain “the poor benefit of a bewildering minute” life slows after such a dinner to a human pace…It is the function of Christmas puddings to restore this indispensable refreshment.”

[Note—Not Necessarily the Current Authors’ Opinions]. The row colours add clarity by clearly separating different aspects or studies within the tables.

### 4.2. Part (ii) Selective Literature Synthesis Regarding Time-Restricted Eating

In the 1930s, an experiment laid the ground for counting calories or calorie-restricted eating (i.e., “how much”) [50]. Later into the last century, a large series of experiments began which researched healthy diets (i.e., “what”) to promote health and prevent disease. Interest into “Meal-Timing, Circadian Rhythms and Life Span” was, *inter alia*, instigated by Halberg and Nelson (Franz Halberg is considered to be the “father of American chronobiology” and a pioneer of the field alongside Pittendrigh and Aschoff) [51]. Since the 2000s, literature concerning meal timing (“when”) and health and disease is increasing in scope and scale. Building on how caloric restriction (including fasting and changed food composition) affects lifespan and health via several hallmarks of aging [52], experiments began to target how limitless provision of the same food coupled with time restriction (namely, random eating versus time-restricted eating) may affect weight and disease.

With regard to the timing of meals, Asher and the late Sassone-Corsi [17] lucidly illustrated the possibly beneficial—or detrimental—interactions between nutrition, metabolism, and circadian clocks. Indeed, from an evolutionary perspective, rather than “how much” and “what” we eat, the “when” we eat seems critical. From such perspective, the dietary paradigm in today’s societies of several meals plus snacks during day and night is inappropriate because our—and other species’—bodies gain natural fitness with daily fasting time windows or time-restricted eating [16]. A 16-h window each day without food is postulated as being good to protect against obesity, cardiovascular diseases, metabolic diseases, and possibly even cancer. There is also suggestion that the corresponding window during which the body’s response to, and the fate of, nutrients is optimal occurs earlier in the day rather than later [53,54].

Importantly, when it comes to timing, researchers do not explore effects of zero diet or fasting, but they are testing a certain form of interval fasting. Panda and colleagues elucidated respective details in several animal experiments: First, the researchers demonstrated that the temporal pattern of eating might determine whether mice eating a diet of high-fat chow are protected from adverse metabolic and hepatic changes despite choosing to consume the same amount of food (calories) [55]. While one group could feed only within an 8-h window [Time-Restricted Feeding or TRF—a slight distinction with “eating” for humans and “feeding” for other animals], the other group was free to feed during day and night. After 18 weeks of the experiment, those mice under high fat chow and TRF weighed 28% less than their counterparts. The extra weight in the mice with *ad lib* access to high fat chow (i.e., across all 24-h of the day) is attributable to increased fat deposits. Indeed, the weight of the mice under high fat chow and TRF was more comparable to mice on a regular chow diet. The same was true for markers of metabolic homeostasis. Furthermore, hepatic steatosis and liver damage were observed in the group eating around the clock but much less so in the TRF group. This is in addition to increased markers of inflammation. Next, experiments tested effects of less stringent TRF window durations against high fat chow diet associated weight gain [56]: Identifying larger temporal windows of food access effective against nutritional challenges (i.e., the temporal boundaries of beneficial TRF benefits) is key to translate observations into practice within a modern work schedule. To this end, allowing access to a high-fat diet for 9 h, 12 h, 15 h, or 24 h evinced that longer daily windows of feeding opportunity led to larger increases in body weight. The lasting or legacy effects of TRF interrupted with *ad lib* feeding were also remarkable. Mice switching between 5 days of TRF and 2 days of *ad lib* (weekday-weekend difference) for 12 weeks presented with a 29% gain in body weight compared a 61% gain in body weight in *ad lib* only mice. Yet, switching from 13 weeks with beneficial TRF to 12 weeks of *ad lib* evinced a “rebound effect” insofar as the mice with 13 weeks TRF put on as much weight as those mice maintained on *ad lib* feeding for the entire 25 weeks (i.e., they “caught up”). This highlights an additional temporal component to intermittent dietary practices. Lastly, mice switched from high fat chow *ad lib* feeding to TRF after 13 weeks or 26 weeks dropped weight in the following 12 weeks. It is important to stress that TRF groups consumed equivalent calories to *ad lib* feeding groups throughout. In addition, these larger effects on body weight were observed for mice fed a high fat chow diet only. No clear effects were observed for regular chow diet TRF vs. regular chow diet *ad lib* conditions—likely due to regular chow diet mice not exhibiting the same extended window of consumption pattern as their high fat chow counterparts under *ad lib* conditions.

That humans eat during very extended time windows is rarely assessed but was empirically evinced by Panda and colleagues who followed 156 healthy and human subjects with no shift-work [57]. Information from a mobile app showed that most ate both frequently and erratically during wakeful hours (half the group consumed their daily dietary intake for 15 h or longer) and that overnight fasting duration coincided with sleep duration. Tendencies to eat late and “metabolic jetlag” [57] due to varying weekday/weekend eating patterns were observed that were similar to jetlag associated with trans-meridian travel. Reducing the time window for eating to 10–11 h/day allowed overweight individuals to reduce body weight, improve sleep, and to feel more energetic.

## 5. Discussion

Christmas time excess and New Year resolutions are a regular practice. The psychology of such practice is hard to shake.

Part (i) suggests that the festive days can have detrimental nutritional effects (Table 2). While there is certainly room for making nutritional habits towards the end of the year safer, we may want to focus on resolutions for the time to come. Psychologically, since many or most people will eat too much and wrong things over the festive days, willingness to consider change regarding eating habits thereafter should be exploited. Consistency in healthy practice may be important to prevent rebound effects making the situation worse [32,56], albeit short-term intermittent *ad lib* eating may not be too detrimental according to the experimental data from high fat diet mice [56]. Successful public health policy [40] that evolves into a social norm [25] and aiding individual health perceptions [25] may all prove beneficial.

Part (ii) points to a comparably simple, and possibly sustainable, change of eating. Indeed, dietary promotion of health and prevention of disease may come from chronobiology or circadian science. More generally, many metabolic pathways are controlled by circadian clocks and follow circadian rhythms [58,59]. Hypothetically, a normal(ized) cyclical expression of metabolic regulators leads to efficient metabolism while compromised temporal expressions are detrimental and may lead to weight gain and associated detrimental facets. Animal studies regarding TRF appear promising: Relatively minor changes towards a maximum of ten hours for eating may yield weight loss effects. Moreover, humans consuming foods that are more nutritious may still benefit from TRF even though there was little benefit of TRF for regular chow fed mice: Indeed, the regular chow fed mice restrict the timing of their consumption to a shorter time window during *ad lib* access anyway. Humans, regardless of the type and volume of food that they consume, may still eat within larger time windows of the day. Importantly, TRE [Time-Restricted Eating or TRE as the human equivalent to Time-Restricted Feeding or TRF] may be effective against both developing obesity and against existing obesity. From studies in animal models, there are lasting effects upon cessation, and TRE remains effective even when applied only 5 days temporarily interrupted by *ad lib* food access during weekends.

Clearly, though, we lack empirical data from human studies. One such study recently concluded that TRE was not more effective than restricting daily calories alone regarding a reduction in body weight, body fat, or metabolic risk factors in obese patients [60]. Yet, an editorial [61] suggested that the relatively small trial’s results are compatible with the possibility of a clinically relevant benefit of TRE. Moreover, persons with longer habitual time windows for eating are likely to benefit most from TRE—in the event, the eating window was reduced from an average of some 10 h at baseline across the 139 study individuals to 8 h [61]. Clearly, more such research is needed and planned already. A feasibility and effects study of 10-h time-restricted eating in career firefighters doing 24-h shift work is under way [62].

Overall, and even though humans are not big mice, why not consider what rodent experiments strongly suggest during Christmas and put this on resolution lists after the festive days? Clearly, the possible benefit could be large. Equally clearly, if in any doubt regarding harm associated with TRE or if you already have medical dietary requirements or practices (e.g., with disease such as diabetes [63]), ask your doctors for advice. In any case, let us not underestimate how detrimental nutritional excesses over Christmas may encourage individuals to tackle their eating habits. Basing such resolutions on circadian science can be promising because they are—in their simplest form [“when to eat”]—easy and possibly sustainable. Of course, paying attention to calorie intake and food composition makes additional sense.

## 6. Conclusions

In conclusion, the Pittendrigh–Aschoff-Christmas-card brings Christmas, chronobiology, and New Year resolutions and health together: The motto “Nicht Immer Arbeiten!” implying “Everything needs its time” could be extended to “eating needs its time”.

## Figures and Tables

**Table 1:** Search string & Inclusion/Exclusion Criteria.

Search String	(“Christmas” [tiab] OR “xmas” [tiab] OR “Festive Season” [tiab] OR “New Year” [tiab] OR “Hogmanay” [tiab] OR “Winter Holiday” [tiab] OR “Winter Break” [tiab] OR “Yule” [tiab] OR “Noel” [tiab]) AND (Nutrition OR Diet OR Food OR Meal OR Drink OR Eat OR Feed)
**Inclusion Criteria**	Language: English or German Population: Humans Exposure/topic: Dietary practice (including characteristics such as type, timing, and/or volume of food, food purchase, and dietary management intervention) or markers thereof (incl. anecdotal evidence). Time of year: Christmas and/or Gregorian New Year (or synonyms of this holiday season) must be explicitly mentioned (and explicitly pertaining to the exposure/topic in full text analysis or opinion style articles that were stimulated by the time of year).
**Exclusion Criteria**	Studies based on traffic accidents, hospital admission incidence, or polar expeditions from 100 years ago, or description of alcohol consumption at this time of year. Primary source research of weight change without specific dietary practice details or with only dietary practice speculation. Articles concerning environmental sustainability. Book reviews.

[tiab] indicates restriction to title and abstract fields. The row colours add clarity by clearly separating different aspects or studies within the tables.

## References

[1] Grandjean P. (2016). Paracelsus Revisited: The Dose Concept in a Complex World. Basic Clin. Pharmacol. Toxicol..

[2] Grandjean P., Bellinger D., Bergman A., Cordier S., Davey-Smith G., Eskenazi B., Gee D., Gray K., Hanson M., van den Hazel P. (2008). The faroes statement: Human health effects of developmental exposure to chemicals in our environment. Basic Clin. Pharmacol. Toxicol..

[3] Lemmer B. (2017). The Circadian Clock is now Ticking even in Stockholm Nobel Prize for the Circadian Rhythms. Deut. Med. Wochenschr..

[4] Menaker M., Colin S. (1996). Pittendrigh (1918-96). Nature.

[5] Daan S., Gwinner E. (1998). Jurgen Aschoff (1913-98). Nature.

[6] Aschoff J. (1951). Die 24-Stunden-Periodik der Maus unter konstanten Umgebungsbedingungen. Die Nat..

[7] Aschoff J. (1954). Zeitgeber der tierischen Tagesperiodik. Die Nat..

[8] Lewis P., Foster R.G., Erren T.C. (2018). Ticking time bomb? High time for chronobiological research. EMBO Rep..

[9] Pittendrigh C.S. (1967). Circadian systems. I. The driving oscillation and its assay in Drosophila pseudoobscura. Proc. Natl. Acad. Sci. USA.

[10] Pittendrigh C.S. (1954). On Temperature Independence in the Clock System Controlling Emergence Time in Drosophila. Proc. Natl. Acad. Sci. USA.

[11] Pittendrigh C.S., Minis D.H. (1972). Circadian systems: Longevity as a function of circadian resonance in Drosophila melanogaster. Proc. Natl. Acad. Sci. USA.

[12] Rosbash M. The Circadian Clock, Transcriptional Feedback and the Regulation of Gene Expression. Nobel Lecture. NobelPrize.org. Nobel Prize Outreach AB 2022. https://www.nobelprize.org/prizes/medicine/2017/rosbash/lecture/.

[13] Lewis P., Oster H., Korf H.W., Foster R.G., Erren T.C. (2020). Food as a circadian time cue—evidence from human studies. Nat. Rev. Endocrinol..

[14] Asher G., Sassone-Corsi P. (2015). Time for food: The intimate interplay between nutrition, metabolism, and the circadian clock. Cell.

[15] Sahar S., Sassone-Corsi P. (2009). Metabolism and cancer: The circadian clock connection. Nat. Rev. Cancer.

[16] Mattson M.P., Allison D.B., Fontana L., Harvie M., Longo V.D., Malaisse W.J., Mosley M., Notterpek L., Ravussin E., Scheer F.A. (2014). Meal frequency and timing in health and disease. Proc. Natl. Acad. Sci. USA.

[17] Verdin E. (2020). Paolo Sassone-Corsi (1956–2020). Science.

[18] McCorristine S., Mocellin J.S.P. (2016). Christmas at the Poles: Emotions, food, and festivities on polar expeditions, 1818–1912. Polar Rec..

[19] Cherchye L., De Rock B., Griffith R., O’Connell M., Smith K., Vermeulen F. (2020). A new year, a new you? Within-individual variation in food purchases. Eur. Econ. Rev..

[20] Pope L., Hanks A.S., Just D.R., Wansink B. (2014). New Year’s Res-Illusions: Food Shopping in the New Year Competes with Healthy Intentions. PLoS ONE.

[21] Bhutani S., Wells N., Finlayson G., Schoeller D.A. (2020). Change in eating pattern as a contributor to energy intake and weight gain during the winter holiday period in obese adults. Int. J. Obes..

[22] Wagner D.R., Larson J.N., Wengreen H. (2012). Weight and body composition change over a six-week holiday period. Eat. Weight Disord.-Stud. Anorex..

[23] Olsson A., Thoren I., Mohammad M.A., Rylance R., Platonov P.G., Sparv D., Erlinge D. (2021). Christmas holiday triggers of myocardial infarction. Scand. Cardiovasc. J..

[24] Lenti M.V., Klersy C., Brera A.S., Musella V., Benedetti I., Padovini L., Ciola M., Croce G., Ballesio A., Gorgone M.F. (2020). Clinical complexity and hospital admissions in the December holiday period. PLoS ONE.

[25] Kadhim N., Amiot C.E., Louis W.R. (2021). The Buffering Role of Social Norms for Unhealthy Eating Before, During, and After the Christmas Holidays: A Longitudinal Study. Group Dyn.-Theory Res. Pract..

[26] Neighbors C., Atkins D.C., Lewis M.A., Lee C.M., Kaysen D., Mittmann A., Fossos N., Rodriguez L.M. (2011). Event-Specific Drinking Among College Students. Psychol. Addict. Behav..

[27] Sarri K., Bertsias G., Linardakis M., Tsibinos G., Tzanakis N., Kafatos A. (2009). The Effect of Periodic Vegetarianism on Serum Retinol and alpha-tocopherol Levels. Int. J. Vitam. Nutr. Res..

[28] Sarri K., Linardakis M., Codrington C., Kafatos A. (2007). Does the periodic vegetarianism of Greek Orthodox Christians benefit blood pressure?. Prev. Med..

[29] Sarri K.O., Linardakis M.K., Bervanaki F.N., Tzanakis N.E., Kafatos A.G. (2004). Greek Orthodox fasting rituals: A hidden characteristic of the Mediterranean diet of Crete. Br. J. Nutr..

[30] Sarri K.O., Tzanakis N.E., Linardakis M.K., Mamalakis G.D., Kafatos A.G. (2003). Effects of Greek orthodox Christian church fasting on serum lipids and obesity. BMC Public Health.

[31] Hirsh S.P., Pons M., Joyal S.V., Swick A.G. (2019). Avoiding holiday seasonal weight gain with nutrient-supported intermittent energy restriction: A pilot study. J. Nutr. Sci..

[32] Wilson M.G., Padilla H.M., Meng L., Daniel C.N. (2019). Impact of a workplace holiday weight gain prevention program. Nutr. Health.

[33] Parker H.L., Curcic J., Heinrich H., Sauter M., Hollenstein M., Schwizer W., Savarino E., Fox M. (2017). What to eat and drink in the festive season: A pan-European, observational, cross-sectional study. Eur. J. Gastroenterol. Hepatol..

[34] Cowley A.J., Stainer K., Murphy D.T., Murphy J., Hampton J.R. (1986). A non-invasive method for measuring cardiac output: The effect of Christmas lunch. Lancet.

[35] Page M.J., McKenzie J.E., Bossuyt P.M., Boutron I., Hoffmann T.C., Mulrow C.D., Shamseer L., Tetzlaff J.M., Akl E.A., Brennan S.E. (2021). The PRISMA 2020 statement: An updated guideline for reporting systematic reviews. BMJ (Clin. Res. Ed.).

[36] Zorbas C., Reeve E., Naughton S., Batis C., Whelan J., Waqa G., Bell C. (2020). The Relationship Between Feasting Periods and Weight Gain: A Systematic Scoping Review. Curr. Obes. Rep..

[37] Harris M. (2003). Ringing in the new year with the SNAP behavioural risk factors in general practice. Aust. Fam. Physician.

[38] Garrow J. (2000). Christmas factor and snacking. Lancet.

[39] Brendieck-Worm C. (2017). Cinnamon—Not Just for Christmas: An Overview of the Use of Cinnamon as a Spice and Herbal Remedy. Z. Ganzheitl. Tiermed..

[40] Yeomans H. (2019). New Year, New You: A qualitative study of Dry January, self-formation and positive regulation. Drug-Educ. Prev. Policy.

[41] Bates J. (2016). Add some spice to ease creaking joints. Nurs. Stand..

[42] Eagle K. (2012). Hypothesis Holiday sudden cardiac death: Food and alcohol inhibition of SULT1A enzymes as a precipitant. J. Appl. Toxicol..

[43] Kloner R.A. (2004). The “Merry Christmas Coronary” and “Happy New Year Heart Attack” phenomenon. Circulation.

[44] Hendry J. (1987). Cardiac output and Christmas lunch. Lancet.

[45] Griffith M.J. (1995). A New Year toast...to the cardioprotective effects of alcohol. Br. Heart J..

[46] (1973). Editorial: The Christmas pudding. Br. Med. J..

[47] Clark N. (1998). A nutritious new year: Resolutions from a to z. Physician Sportsmed..

[48] Cannon G. (2006). Out of the christmas box. Public Health Nutr..

[49] Cannon G. (2004). Out of the Christmas box. Public Health Nutr..

[50] McCay C.M., Crowell M.F., Maynard L.A. (1935). The effect of retarded growth upon the length of life span and upon the ultimate body size. J. Nutr..

[51] Nelson W., Halberg F. (1986). Meal-timing, circadian rhythms and life span of mice. J. Nutr..

[52] Acosta-Rodriguez V.A., Rijo-Ferreira F., Green C.B., Takahashi J.S. (2021). Importance of circadian timing for aging and longevity. Nat. Commun..

[53] Sutton E.F., Beyl R., Early K.S., Cefalu W.T., Ravussin E., Peterson C.M. (2018). Early Time-Restricted Feeding Improves Insulin Sensitivity, Blood Pressure, and Oxidative Stress Even without Weight Loss in Men with Prediabetes. Cell Metab..

[54] Xie Z., Sun Y., Ye Y., Hu D., Zhang H., He Z., Zhao H., Yang H., Mao Y. (2022). Randomized controlled trial for time-restricted eating in healthy volunteers without obesity. Nat. Commun..

[55] Hatori M., Vollmers C., Zarrinpar A., DiTacchio L., Bushong E.A., Gill S., Leblanc M., Chaix A., Joens M., Fitzpatrick J.A. (2012). Time-restricted feeding without reducing caloric intake prevents metabolic diseases in mice fed a high-fat diet. Cell Metab..

[56] Chaix A., Zarrinpar A., Miu P., Panda S. (2014). Time-restricted feeding is a preventative and therapeutic intervention against diverse nutritional challenges. Cell Metab..

[57] Gill S., Panda S. (2015). A Smartphone App Reveals Erratic Diurnal Eating Patterns in Humans that Can Be Modulated for Health Benefits. Cell Metab..

[58] Panda S., Antoch M.P., Miller B.H., Su A.I., Schook A.B., Straume M., Schultz P.G., Kay S.A., Takahashi J.S., Hogenesch J.B. (2002). Coordinated transcription of key pathways in the mouse by the circadian clock. Cell.

[59] Gamble K.L., Berry R., Frank S.J., Young M.E. (2014). Circadian clock control of endocrine factors. Nat. Rev. Endocrinol..

[60] Liu D., Huang Y., Huang C., Yang S., Wei X., Zhang P., Guo D., Lin J., Xu B., Li C. (2022). Calorie Restriction with or without Time-Restricted Eating in Weight Loss. N. Engl. J. Med..

[61] Laferrere B., Panda S. (2022). Calorie and Time Restriction in Weight Loss. N. Engl. J. Med..

[62] Manoogian E.N.C., Zadourian A., Lo H.C., Gutierrez N.R., Shoghi A., Rosander A., Pazargadi A., Wang X., Fleischer J.G., Golshan S. (2021). Protocol for a randomised controlled trial on the feasibility and effects of 10-hour time-restricted eating on cardiometabolic disease risk among career firefighters doing 24-hour shift work: The Healthy Heroes Study. BMJ Open.

[63] Jakubowicz D., Landau Z., Tsameret S., Wainstein J., Raz I., Ahren B., Chapnik N., Barnea M., Ganz T., Menaged M. (2019). Reduction in Glycated Hemoglobin and Daily Insulin Dose Alongside Circadian Clock Upregulation in Patients With Type 2 Diabetes Consuming a Three-Meal Diet: A Randomized Clinical Trial. Diabetes Care.

